# Nucleofection of Adipose Mesenchymal Stem/Stromal Cells: Improved Transfection Efficiency for GMP Grade Applications

**DOI:** 10.3390/cells10123412

**Published:** 2021-12-03

**Authors:** Francesco Agostini, Carla Vicinanza, Gianni Biolo, Paola Spessotto, Francesco Da Ros, Elisabetta Lombardi, Cristina Durante, Mario Mazzucato

**Affiliations:** 1 Stem Cell Unit, Centro di Riferimento Oncologico di Aviano (CRO) IRCCS, Via F. Gallini 2, 33081 Aviano, Italy; carla.vicinanza@cro.it (C.V.); fdaros@cro.it (F.D.R.); elombardi@cro.it (E.L.); cdurante@cro.it (C.D.); mmazzucato@cro.it (M.M.); 2 Unit of Internal Medicine, Clinica Medica, Department of Medical Surgical and Health Sciences, University of Trieste, Strada di Fiume 447, 34100 Trieste, Italy; biolo@units.it; 3 Division of Molecular Oncology, Centro di Riferimento Oncologico di Aviano (CRO) IRCCS, Via F. Gallini 2, 33081 Aviano, Italy; pspessotto@cro.it

**Keywords:** nucleofection, non-viral genetic modification, mesenchymal stem cells, Good Manufacturing Practice, supernatant rich in growth factors, lipid raft, membrane composition

## Abstract

Nucleofection (NF) is a safe, non-viral transfection method, compatible with Good Manufacturing Practice guidelines. Such a technique is useful to improve therapeutic effectiveness of adipose tissue mesenchymal stem cells (ASC) in clinical settings, but improvement of NF efficiency is mandatory. Supernatant rich in growth factors (SRGF) is a clinical-grade medium additive for ASC expansion. We showed a dramatically increased NF efficiency and post-transfection viability in ASC expanded in presence of SRGF (vs. fetal bovine serum). SRGF expanded ASC were characterized by increased vesicle endocytosis but lower phagocytosis properties. SRGF increased n-6/n-3 ratio, reduced membrane lipid raft occurrence, and lowered intracellular actin content in ASC. A statistical correlation between NF efficiency and lipid raft availability on cell membranes was shown, even though a direct relationship could not be demonstrated: attempts to selectively modulate lipid rafts levels were, in fact, limited by technical constraints. In conclusion, we reported for the first time that tuning clinical-grade compatible cell culture conditions can significantly improve ASC transfection efficiency by a non-viral and safe approach. A deep mechanistic characterization is extremely complex, but we can hypothesize that integrated changes in membrane structure and intracellular actin content could contribute to explain SRGF impact on ASC NF efficiency.

## 1. Introduction

Mesenchymal stem/stromal cells (MSC) are multipotent adult stem cells that can be obtained from bone marrow [1] as well as, among other sources, from adipose tissue (adipose-derived mesenchymal stem/stromal cells, ASC) [2]. The capacity to downregulate immune response of the organism and their differentiation capability, together with their homing potential toward inflammatory sites, makes expanded ASC/MSC an ideal Advanced Cell Therapy product for a variety of applications as, e.g., cancer, inflammatory pathologies, and reconstructive medicine [3,4]. Safety issues require careful consideration, also in relation to clinical effectiveness, before stem cell therapy application to human patients. Previous works and a recent meta-analysis reported ASC could be applied in controlled clinical trials without inducing serious adverse events in recipients [5,6]; nevertheless, follow up results regarding long term safety of patients are presently lacking. Moreover, standardization of experimental procedures to obtain ASC/MSC based cell therapy products are required to improve their safety and clinical effectiveness [7].

To obtain clinically relevant cell amounts, ASC/MSC must be ex vivo expanded in compliance with Good Manufacturing Practice (GMP) guidelines [8]. Fetal bovine serum (FBS) is a frequently used medium additive, but, as stated by the Committee for medicinal products for human use, it is not recommended for production of ASC/MSC as Advanced Cell Therapy product [9], as it is derived from animal origin. We previously developed Supernatant Rich in Growth Factor (SRGF) [10], i.e., a growth factor mixture obtained by CaCl_2_ addition to platelet concentrates collected by platelet apheresis from healthy donors. We showed that SRGF can be an optimal FBS substitute accelerating ASC growth rate without affecting cell potency, identity, and karyotype stability [11]. SRGF was shown, in vitro, to upregulate ASC homing capacity on selected tumor types, suggesting that such medium additive can affect relevant cellular functions for potential therapeutic applications [12].

Genetic engineering can strikingly enhance or tailor the therapeutic potential of ASC/MSC [13], in turn improving the balance between clinical effectiveness and potential undesired effects exerted on patients. Gene delivery methods involving viral cell infection are highly efficient, but this approach can be potentially harmful for clinical applications due to uncontrolled gene integration in the host cell genome and to systemic immune response in the patient [14]. Non-viral methods delivering exogenous genes to expanded cells can strongly reduce risks for patients, but they are poorly efficient when applied on ASC/MSC [15]. Nucleofection (NF) is one of the widely used gene transfection methods that is based on cell exposure to appropriate electric fields; this induces the formation of transient pores on the cell membrane, in turn allowing DNA to enter in the cell by passive diffusion [16,17]. At present, little is known about complex mechanisms leading to pore formation in the outer membrane of a living cell [18,19]. Membrane fluidity could affect pore formation on cell surface after electric pulse [20], but such evidence was previously challenged [21,22]. Saturated and unsaturated (for, e.g., n-3 or n-6) fatty acid distribution within the cell membrane can affect membrane deformability and stiffness, in turn affecting cell properties [23]. Moreover, within NF, pore formation in cell membrane is likely to occur in liquid-disoriented regions of the cell membrane, i.e., out of the so called lipid rafts [20]. Lipid rafts are ordered and dense clusters of sterol- and sphingolypid-enriched domains, characterizing specific regions of the outer cell membrane [24]. Previous publications demonstrated that an increase in selected n-3 fatty acids, such as Docosahexaenoic acid (DHA), upregulated and stabilized availability of lipid rafts in the cell membrane [25,26].

Fatty acid composition of the cell membrane can potentially modulate other cellular routes of internalization, i.e., endocytosis or uptake of solid nanoparticles by phagocytosis [27,28]. Published works showed that silica nanoparticles are internalized through lipid raft associated phagocytosis [29,30].

Culture conditions can alter cell membrane properties and composition [31,32]. The impact of culture conditions on genetic modification efficiency by NF is poorly investigated. In this work, we primarily evaluated the effect of SRGF on NF efficiency in ASC. To better describe the impact of such medium additive on different cell internalization routes, we also focused our interest on cell endocytosis and phagocytosis processes. Finally, we attempted to identify mechanisms explaining SRGF mediated effect on NF efficiency in our ASC, mainly focusing our attention on changes in membrane composition and lipid raft availability on cell surface.

## 2. Materials and Methods

### 2.1. Cell Isolation, Expansion, and Characterization

Stromal vascular fraction cells were isolated from adipose tissue, applying the protocol we previously published [11]. Lipoaspirates were obtained from female breast cancer patients (45.9 ± 3.0 years) undergoing reconstructive lipofilling (study protocol code: CRO-2016-30; approved on 30th of June 2016). Stromal vascular fraction cells were frozen in autologous serum supplemented with 5% dimethyl sulfoxide [11] until use. ASC were expanded as previously published [12] with few modifications. Thawed stromal vascular fraction cells, derived from *n* = 5 patients (43.1 ± 2.1 years) were separately plated in standard T25 tissue culture flasks (BD Biosciences; Franklin Lakes, NJ, USA) with Minimum Essential Medium Eagle-Alpha Modification (α-MEM) (Lonza; Basel, Switzerland) added with 10% *v*/*v* FBS and, in parallel, with 5% vol/vol SRGF. After 24 h, nonadherent cells were removed, and fresh medium was added after a single wash with phosphate-buffered saline (PBS). Before reaching confluence, cells were detached by trypsin-ethylenediaminetetraacetic acid (EDTA) (TrypLe Select; Life Technologies-Thermo Fisher Scientific, Waltham, MA, USA). Resuspended cells were seeded (at passage P1 and at each following cell passage) and expanded in the presence of 10% *v*/*v* FBS (10% FBS ASC) or different concentrations of SRGF spanning from 1.25% until 20% (as indicated). As stated in a previous work [11], and as per the criteria defined by the International Society of Cell Therapy [33], phenotype and differentiation potential of 10% FBS ASC, and of our 5% SRGF ASC in expanded cells, were shown to be compliant with ASC or MSC. Further, 10% FBS ASC were used from passage P3 until P9, while SRGF ASC from P3 until P13. We previously demonstrated stability of cell proliferation potential at such passages for both FBS and SRGF ASC [11].

Population doubling time (PDT) was calculated as:

PDT = time × ln(2)/ln(total collected cells/total seeded cells)(1)

where “time” is expressed as hours between passages and “ln” is natural logarithm.

### 2.2. Nucleofection

Non-viral transfection procedures were performed by Nucleofector^®^ 2b device (Lonza) following manufacturer’s instructions with modifications. Upon NF, expanded ASC were collected and resuspended in an NF buffer (Ingenio^®^ Electroporation Solution, Mirus Bio Corporation, Madison, WI, USA) (500 × 10^3^ cells/test in 100 μL of buffer). Then, 2 μg of the empty vector pMaxGFP (from Lonza), encoding for the green fluorescent protein (GFP), were added to the mixture. As a control, NF of ASC without DNA was performed. To set up transfection conditions, C-17 or U-23 programs were applied to 10% FBS ASC and to 5% SRGF. At 24 h after shock administration, cell viability and NF efficiency were tested. Cell viability and transfection efficiency were assayed 24 h after NF. In setup experiments, transfection efficiency was assessed using ImageJ software (National Institutes of Health, Bethesda, MD, US) to count total and GFP positive cells in separate images of adhering cells. Images were taken by an inverted fluorescence microscope (Nikon, Tokyo, Japan) equipped by a digital color camera (Motic, Hong Kong, China). Fluorescence imaging parameters (lamp output energy: 100%; exposure time: 300–400 msec; offset: 0.8) were set, in untreated cell samples, in order to totally avoid cell autofluorescence (dark pictures). The number of total adhering cells (brightfield imaging) was used to assess ASC survival after electric shock administration (see Calculations). At least five images (10× objective fields) were analyzed by manual counting of GFP positive cells (by two independent operators). In further experiments, efficiency of NF (by C-17 program) in our ASC expanded in the presence of different SRGF concentrations (20%; 10%; 5%; 2.5%; 1.25%; 0.62%; and 0.15%) was tested by standard cytofluorimetry (FACS Canto II; BD Biosciences) and data were analyzed by Diva Software (BD Biosciences). The ASC population was defined by forward and side scatter physical parameters. Within flow cytometry experiments, to set the threshold gate identifying GFP positive cells, ASC transfected without DNA vector were analyzed as control condition. Evaluation of transfection efficiency by cytofluorimetry was not feasible in NF setup experiments due to the low cell viability associated with the U-23 protocol.

### 2.3. Calculations:

In preliminary NF setup experiments, cell viability was calculated as:

Cell viability = 100 × total adhering ASC/total electroporated ASC(2)

Total adhering ASC values were determined as:

Total adhering ASC = (mean number of adhering ASC in the image/image area) × (culture vessel area)(3)

NF efficiency was determined as:

NF Efficiency = 100 × total GFP positive ASC/total adhering ASC(4)

Total GFP positive ASC values were determined as:

Total GFP positive ASC = (mean number of GFP positive ASC in the image/image area) × culture vessel area(5)

### 2.4. Overexpression of CD63

By C-17 protocol, 5% SRGF and 10% FBS ASC (5 × 10^5^ cells) were nucleofected to deliver with 1 μg of a mammalian expression vector pEGFP C2 (AddGene, Watertown, MA, USA) containing the CD63 gene sequence. To evaluate CD63 NF efficiency, the percent fraction of GFP positive cells was evaluated by flow cytometry: thus, as a control for such experiments, NF of ASC without DNA was performed. Overexpression of CD63 was also evaluated by Western blot analysis. In such experiments, NF of 1 μg of the mammalian expression vector pEGFP C2 (AddGene) containing the CD63 gene sequence was performed. As control for Western blot analysis, NF of the empty pEGFP C2 vector was performed. Then, 24 h after transfection, cells were collected and lysed by NP40 Cell Lysis Buffer (Thermo Fisher Scientific). After separation by Mini Protean TGX Precast Gel (gradient 4–20%) proteins were blotted on nitrocellulose membranes. Expression of CD63 was analyzed by Western blot using anti-human CD63 (Invitrogen, Carlsbad, CA, USA) (dilution 1:1000) and, as loading control, anti-human GAPDH (dilution 1:1000) (Calbiochem, San Diego, CA, USA) antibodies. Intensity of CD63 and GAPDH Western blot bands were quantified by ImageJ software (National Institutes of Health). For comparison between electroporated 10% FBS and 5% SRGF ASC, CD63 expression level was defined as ratio between CD63 and GAPDH band intensity.

### 2.5. Intracellular Vesicle Labelling

Vesicle internalization capacity of ASC was evaluated by exposing cells for 12–18 h to the intracellular vesicle labelling dye FM 1-43 (Thermo Fisher Scientific) at the final concentration of 8 μM in culture medium. As indicated by the manufacturer, internalization of such tracer is very rapid. During prolonged incubation time, a steady state of intracellular vesicle labelling turnover could safely be obtained. Images of living cells were taken by epifluorescence microscopy equipped by digital camera (Nikon Digital Sight DS; Nikon, Tokyo, Japan). Fluorescence cell imaging (20× objective) was performed setting conditions (lamp output energy: 75%; exposure time: 150 msec; digital gain: 1; and offset: default) in untreated cells in order to fully avoid cell autofluorescence (dark pictures). Collected images were analyzed by ImageJ software (National Institutes of Health).

### 2.6. Phagocytosis

Phagocytosis capacity was analyzed exposing cells to 25 μg/mL ultrastable fluorescent silica nanobeads (Merck, Darmstadt, Germany) in complete cell culture medium. Nanobead diameter was 50 nm and 120 nm. Both 50 nm and 120 nm nanobeads were separately resuspended in cell culture medium and were briefly sonicated (Sonoplus; BANDELIN electronic GmbH & Co, Berlin, Germany) in wet ice to disrupt macro-aggregates. After 24 h exposure to nanobeads, cells cultured on cover glass slides were labelled with 10 μM Carboxyfluorescein Diacetate, Succinimidyl Ester (CFDA-SE) (Thermo Fisher Scientific) and thoroughly washed by PBS (three times). Cover slides were then mounted on glass microscope slides and immediately analyzed. Availability of phagocyted nanobeads within fluorescently labelled cells was immediately analyzed by epifluorescence microscopy equipped by digital camera (Nikon Digital Sight DS;). Fluorescence cell imaging (10× objective) was performed setting conditions (lamp output energy: 75%; exposure time: 300 msec-green channel for CFDA-SE, 120 msec-red channel for nanobeads; digital gain: 1; and offset: default) in untreated cells in order to fully avoid cell autofluorescence (dark pictures).

### 2.7. Supplementation of Expanded ASC

In order to supplement selected fatty acids in cell culture media, 10% FBS ASC, 5% SRGF, and 1.25% SRGF ASC were expanded for 24 h in the presence of commercial customized fatty acid blends named ACD3 and ACD4 solutions (Remembrane, Bologna, Italy). ACD3 contained the following Omega-6 fatty acids: linoleic acid (33.3%); dihomo-gamma-linolenic acid (33.3%); and arachidonic acid (33.3%). ACD4 contained the following Omega-3 fatty acids: alpha-Linolenic acid (33.3%); eicosapentaenoic acid (33.3%); and docosahexaenoic acid (33.3%). ACD3 and ACD4 were used at 1:500 dilution to supplement 10% FBS and 5% SRGF ASC, while 1.25% SRGF ASC were supplemented at 1:1000 dilution. Exposure of 1.25% SRGF ASC to ACD3 and ACD4 at 1:500 dilution caused cell toxicity (data not shown) demonstrated by morphological modifications. Prolongation of ACD3 and ACD4 supplementation in our ASC at 48 h also induced cell toxicity. Furthermore, 5% SRGF ASC were supplemented by adding DHA (Remembrane) for 72 h at final dilutions of 1:250 and 1:500. Reagent concentrations in original fatty acid supplementation products were not disclosed by the manufacturer. For longer term lipid raft depletion, expanded 10% FBS ASC were exposed to lower MCD concentrations. MCD dilutions (150 μM; 300 μM; and 600 μM) that failed to induce cell morphological alterations were selected as appropriate low toxicity assay conditions.

### 2.8. ASC Membrane Composition

Membrane fatty acid composition of 10% FBS ASC and of 5% and 1.25% SRGF ASC was analyzed by gas-chromatography–flame ionization detection (GC-FID) as previously published [34]. After complete cell lysis, total lipid extraction was performed in a chloroform–methanol (2:1) solution, containing 50 mg/L of butylhydroxytoluene (Sigma–Aldrich, Inc, St. Louis, MO, USA) as antioxidant, and NaCl. After centrifugation, the lower lipid phase was collected and dried under nitrogen flux at 40 °C. Pellets were dissolved in toluene and, after the addition of 2% H_2_SO_4_ in methanol, samples were heated at 50 °C for 2 h. After neutralization, fatty acid methyl esters were extracted by hexane addition and collected under nitrogen flux. Lipids were separated and identified by GC-FID (GC 6850; Agilent Technologies, Santa Clara, CA, USA). Helium was used as carrier gas. Chromatographic conditions and utilized fatty acid standards were exactly as previously published [34].

### 2.9. Labelling and Quantification of Lipid Rafts on Cell Membrane

Occurrence of lipid rafts on ASC membranes was assessed taking advantage of the commercially available Vybrant^®^ Lipid Raft Labeling Kit-Alexa Fluor 555 (Molecular Probes–Thermo Fisher Scientific, Waltham, MA, USA). The assay is based on the capacity of subunit B of cholera toxin to bind the pentasaccharide chain of plasma membrane ganglioside GM1, which selectively segregates into lipid rafts. The staining procedure was performed following the manufacturer’s protocol for cells adhering on a coverslip. A three-dimensional scan of labeled cells was performed by the TCS-SP8 Confocal System (Leica Microsystems) interfaced with the Leica Application Suite software. Confocal cell imaging (Objective: 63× PlanApo CS2/1.4 oil) was performed setting conditions (Pinhole set: 1; Diode DPSS 561 emission: 18.6%; and Photomultiplier HyD3 gain: 25%) in untreated cells, in order to fully avoid cell autofluorescence (dark pictures). Random image fields were recorded for each experimental condition. Signal specificity was demonstrated by treating 10% FBS ASC with Methyl-β-cyclodextrin (MCD, Sigma) a cholesterol sequestrating agent that can deplete lipid rafts from cell membranes [35]. For such purposes, MCD was used at the final sublethal concentration of 5 mM for 15 min. By ImageJ software (National Institutes of Health), mean fluorescence intensity was evaluated in each image of the collected z-stack in manually defined regions of interest.

### 2.10. Intracellular Actin Labelling

In a subset of samples, at the end of the lipid raft labelling procedure, the intracellular actin staining procedure was performed. Briefly, cells were fixed by cold 4% paraformaldehyde in PBS for 20 min. After membrane permeabilization by 0.1% Triton X-100, coverglasses were incubated with PBS containing 2% bovine serum albumin as unspecific site blocking agent and, in turn, with Alexa Fluor 633 phalloidin (Thermo Fisher Scientific) (dilution 1:400) as total actin labelling agent. After 3 washes by 0.1% Tween in PBS coverslips were mounted using glycerol and stored at +4 °C until analysis by confocal microscopy. A three-dimensional scan of labeled cells was performed by the TCS-SP8 Confocal System (Leica Microsystems) interfaced with the Leica Application Suite software. Confocal cell imaging settings were as above. By ImageJ software (National Institutes of Health), mean fluorescence intensity was evaluated in each image of the collected z-stack in manually defined regions of interest.

### 2.11. Total and Membrane Protein Analysis

Total and membrane protein fractions were isolated by a previously published method [36] with minor adaptations. Briefly, 10% FBS and 5% SRGF ASC (1 × 10^6^ cells) were plated in separate Petri dishes (BD Biosciences) and left overnight in a standard cell culture incubator. Then, cells were washed twice with ice-cold PBS and thereafter incubated (30 min in ice) with 8 mL PBS containing 0.25 mg/mL EZ-Link Sulfo-NHS-SS Biotin (Thermo Fisher Scientific). Biotinilation was stopped by adding 450 μL of 50 mM Tris (pH 8). Cells, collected by mechanical scraping, were centrifuged at 500× *g* for 5 min at 4 °C. The cell pellets were washed with 5 mL Tris-buffered saline (25 mM Tris, 0.15 M sodium chloride, pH 7.2) and further resuspended in 400 μL lysis buffer (25 mM Tris-HCl, 50 mM NaCl, 0.5% (*w*/*v*) DOC and 0.5% (*w*/*v*) Triton X-100) containing protease inhibitors (Roche). After repeated forcing through a 21 G gauge needle, lysates were incubated 30 min in ice and centrifuged at 14,000× *g* for 2 min. Small aliquots (50 μL) of such total protein extract were added with Laemli sample buffer (BioRad; Hercules, CA, USA) added with 2-Mercaptoethanol (10% *v*/*v*) and stored at −80 °C until analysis. Neutravidin Agarose beads (100 mL, Thermo Scientific) were incubated with the remaining volume of supernatants in gently rocking conditions at room temperature. After sequential washing with PBS, 0.5% (*w*/*v*) Triton X-100, 0.05% (*w*/*v*) DOC and 500 mM NaCl, 125 mM Tris, 10 mM EDTA, and 0.5% (*w*/*v*) Triton X-100 (pH 8), cell surface proteins were eluted from the Neutravidin beads with Laemli sample buffer (BioRad). Total and membrane protein extracts were separated by Mini Protean TGX Precast Gel (gradient 4–20%) and blotted on nitrocellulose membranes. Protein band visualization was performed by standard Red ponceau (BioRad) staining.

### 2.12. Statistics

Results are derived from experimental tests performed using ASC separately isolated from *n* = 5 patients and expanded by selected culture protocols. Mean reported values were calculated from experiments repeated at least three (i.e., three to five) times, using cells from different patients but pair-matching results obtained from the same patient. Data are presented as the mean ± S.E.M. Statistical significance of differences between mean cell viability and electroporation efficiency measured in 10% FBS and 5% SRGF ASC after electroporation by C-17 and U-23 protocols was assessed by Two-way ANOVA with Tukey’s HSD and Bonferroni’s correction as a post hoc test. Statistical significance of differences between mean NF efficiency in 10% FBS ASC was compared to 5% SRGF ASC and in turn to 20%, 10%, 2.5%, 1.25%, 0.62%, and 0.15% SRGF ASC by One-way ANOVA with Tukey’s HSD and Bonferroni’s correction as a post hoc test. Student’s *t*-test for paired data was applied to assess difference significance between mean electroporation efficiency measured in 10% FBS and 5% SRGF ASC after transfection of CD63 expression vector. Similarly, significance of CD63 expression level difference after overexpression in 10% FBS and 5% SRGF ASC, assessed by Western blot band quantification, was assayed by Student’s *t*-test for paired data. Two-way ANOVA with Tukey’s HSD and Bonferroni’s correction as a post hoc test was also applied to analyze significant differences between fatty acid membrane composition parameters in non-supplemented and ACD3 or ACD4 supplemented ASC. Similarly, Two-way ANOVA with Tukey’s HSD and Bonferroni’s correction as a post hoc test was applied to analyze significant differences between NF efficiency and nanoparticle and FM1-43 uptake of non-supplemented and ACD3 or ACD4 supplemented ASC. One-way ANOVA with Tukey’s HSD was used to assess significance between mean fluorescence intensity measured in 10% FBS and 5% or 1.25% SRGF ASC after labelling lipid rafts or total intracellular actin. Effect of MCD or DHA was assessed by One-way ANOVA with Tukey’s HSD, limiting the analysis to 10% FBS and 5% SRGF ASC, respectively. Student’s *t*-test was applied, as appropriate, for post hoc analysis.

## 3. Results

### 3.1. SRGF Expanded ASC Can Be Efficiently Nucleoporated by the C-17 Program

**Figure 1:**
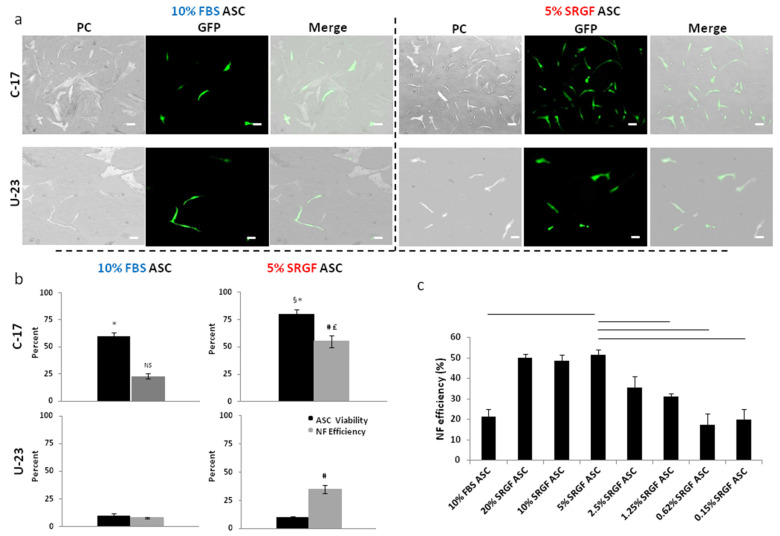
(**a**) shows representative images of 10% FBS and 5% SRGF ASC taken after NF performed by C-17 and U-23 electric pulse administration (protocol setup). Cell viability and transfection efficiency shown in (**b**) were calculated by image analysis as described in Methods. *, *p* < 0.001 vs. U-23 program; §, *p* < 0.01 vs. 10% FBS ASC; #, *p* < 0.01 vs. 10% FBS ASC; £, *p* < 0.05 vs. U-23 program; NS, not significantly different from U-23 program. Scale bar, 25 μm. (**c**) reports transfection efficiency, evaluated by flow cytofluorimetric approach, after NF by C-17 program of ASC expanded in presence of 10% FBS and of different concentrations of SRGF (from 20% to 0.15%, as indicated). The percent fraction of GFP positive cells after NF without DNA (control condition) of both 10% FBS and 5% SRGF ASC was close to zero (data not shown). Solid lines connect significantly (*p* < 0.01) different mean values. In particular, by One-way ANOVA with Tukey’s HSD and Bonferroni’s correction as a post hoc test, NF efficiency in 10% FBS ASC was compared to 5% SRGF ASC. Significance of differences between NF efficiency measured in 5% SRGF ASC was assessed by the same test, also in comparison with mean values obtained in ASC expanded in presence of indicated SRGF concentrations. Reported images and mean (quantification) values represent results derived from at least three independent experimental tests.

To set up the NF experimental protocol, the pMaxGFP vector was delivered to ASC expanded in standard culture conditions, i.e., in presence of 10% *v*/*v* FBS (10% FBS ASC), and to ASC expanded in presence of 5% SRGF (5% SRGF ASC). Cell viability and NF efficiency (Figure 1a,b) were evaluated after application of two different NF programs (C-17 and U-23).

Using the C-17 program, a significantly (*p* < 0.001) higher cell viability was obtained both in 10% FBS and 5% SRGF ASC, when compared to the U-23 program. Cell viability following application of the U-23 program was strikingly low (<10%) in both 10% FBS and 5% SRGF ASC. Restricting the analysis to the C-17, viability of 5% SRGF ASC after electroporation was significantly (*p* < 0.01) higher vs. 10% FBS ASC. Both NF methods allowed significantly (*p* < 0.01) higher transfection efficiency in 5% SRGF ASC than in 10% FBS ASC but, strikingly, the C-17 method induced the highest NF efficiency in 5% SRGF ASC. Thus, considering together viability and NF efficiency results, C-17 program application to 5% SRGF ASC could warrant the highest yield of GFP expressing cells. Such evidence prompted us to select the C-17 as the appropriate program to transfect our ASC. After a recovery time of almost 24 h after NF by C-17, the mean population doubling time was 38.5 ± 4.6 h in 5% SRGF ASC and 147.9 ± 15.7 h in 10% FBS ASC.

We hypothesized that modulation of SRGF availability in the cell culture medium could tune NF efficiency. By cytofluorimetic assays (Figure 1c), we confirmed that the C-17 protocol induced in 5% SRGF ASC a significantly greater NF efficiency, when compared to 10% FBS. Nevertheless, we showed that NF efficiency evaluated in 10% and 20% SRGF ASC was not significantly different from 5% SRGF ASC. Otherwise, decreased SRGF concentrations at 1.25% or below induced a marked and significant reduction in NF efficiency when compared to 5% SRGF ASC. Notably, no statistically significant difference could be demonstrated when comparing NF efficiency measured in 1.25%, 0.62%, and 0.15% SRGF ASC, indicating 1.25% as the SRGF concentration that mostly reduced NF efficiency.

### 3.2. Method Validation: Overexpression of CD63 Gene

**Figure 2:**
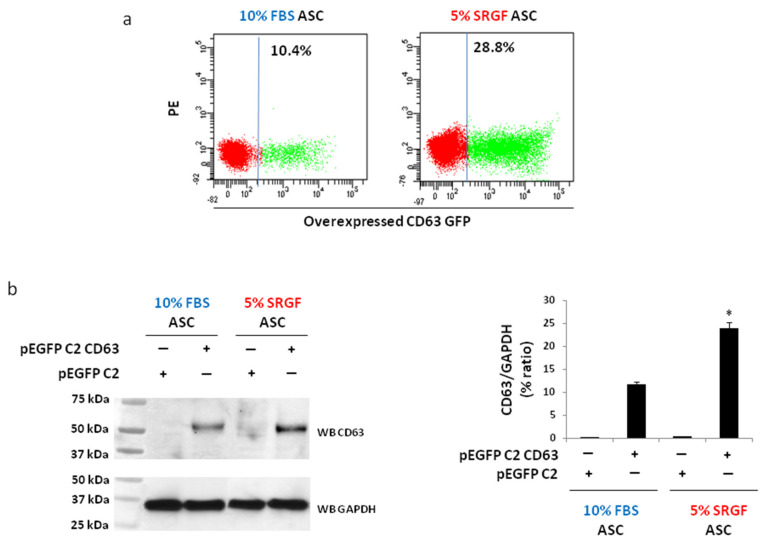
(**a**) reports representative dot plots obtained evaluating GFP positive cells by flow cytometry after NF of a pEGFP C2 vector encoding for CD63 in 10% FBS and 5% SRGF by C17 program. The gating threshold (vertical solid line) identifying GFP negative cells was defined analyzing ASC after NF without DNA vector (as control). (**b**) reports the evaluation by Western blot analysis of CD63 overexpression after NF by C-17 program of a pEGFP C2 vector encoding for CD63. For Western blot analysis, a pEGFP C2 empty vector was transfected (as control condition) in both 10% FBS and 5% SRGF by C17 program. Original unmodified images of analyzed Western blot are reported in Supplementary Figure S1. CD63 band density quantification was expressed as percent ratio to the housekeeping gene GAPDH. *; *p* < 0.01 vs. 10% FBS ASC. Reported images and quantification values represent results derived from at least three independent experimental tests.

To validate our results, we overexpressed a non-toxic gene as CD63. Results reported in Figure 2a showed that NF efficiency of the CD63-GFP expression vector in 5% SRGF ASC was significantly higher when compared to 10% FBS ASC. As evidenced by Western blot analysis (Figure 2b), after NF of the control empty vector, CD63 endogenous expression levels were negligible both in 10% FBS as well as in 5% SRGF ASC. The same analysis (Figure 2b) clearly demonstrated that CD63 expression was significantly (*p* < 0.01) higher in transfected 5% SRGF ASC when compared to 10% FBS ASC.

### 3.3. SRGF Impact on ASC Endocytosis and Phagocyotis Properties

**Figure 3:**
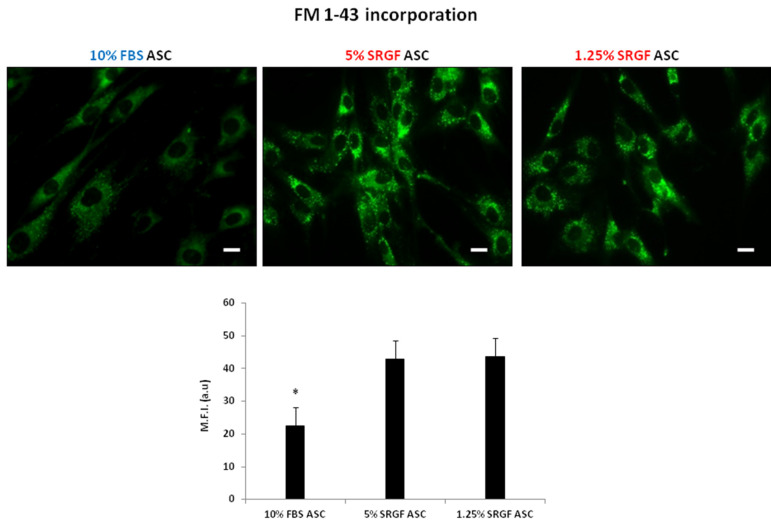
Figure 3 shows FM1-43 uptake efficiency in 10% FBS and 5% SRGF, and 1.25% SRGF ASC. Representative images as well as evaluation of mean fluorescence intensity as arbitrary units [M.F.I. (a.u.)] were reported. *; *p* < 0.01 vs. 5% SRGF ASC and vs. 1.25 % SRGF ASC. Scale bars: 10 μm. Reported images and mean quantification values represent results derived from at least three independent experimental tests.

**Figure 4:**
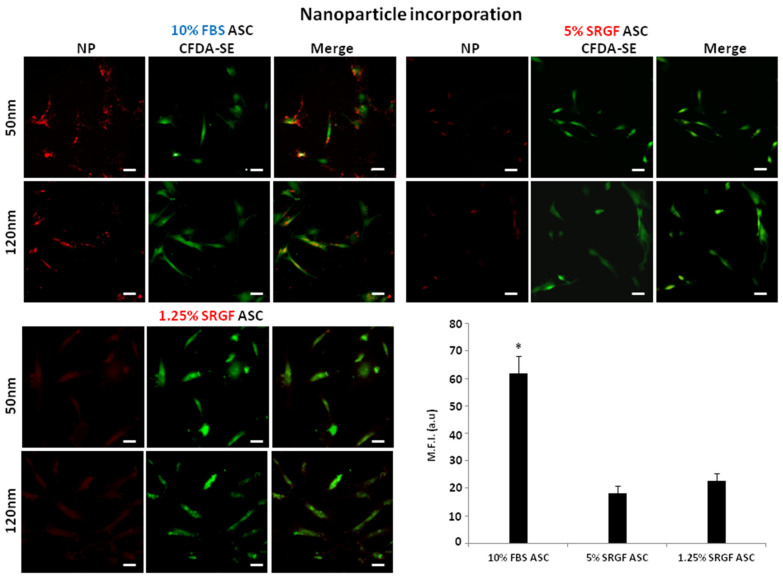
Figure 4 shows fluorescent nanoparticle uptake efficiency in 10% FBS, 5% SRGF and 1.25% SRGF ASC. Both 50 nm and 120 nm beads were administered to expanded ASC labelled by CFDA-SE. Representative images as well as evaluation of mean fluorescence intensity as arbitrary units (M.F.I. (a.u.)) were reported. Scale bars: 30 μm *; *p* < 0.01 vs. 5% SRGF ASC and vs. 1.25 % SRGF ASC. Reported images and mean quantification values represent results derived from at least three independent experimental tests.

Figure 3 shows that 10% FBS ASC were characterized by a significantly (*p* < 0.01) lower capacity to uptake FM 1-43 dye in intracellular vesicles when compared to both 5% and 1.25% SRGF ASC. Otherwise, Figure 4 shows that uptake capacity of both 50 nm and 120 nm nanoparticles by 10% FBS ASC was strikingly higher (*p* < 0.01) when compared to 5% and 1.25% SRGF ASC. Detailed and higher magnification images of ASC exposed to 50 nm and 120 nm nanoparticles are enclosed in Supplementary Figure S2.

### 3.4. SRGF Impact on Membrane Fatty Acid Composition and on Lipid Raft Formation

We hypothesized the abovementioned results were likely to rely on the alteration of cell membrane properties mediated by the different medium additives. For such reasons, in order to investigate the differential impact of SRGF and FBS on ASC, we evaluated by GC-FID, their lipid membrane composition. A detailed description of lipid membrane composition analysis is reported in Supplementary Table S1. We showed that the fraction of pooled saturated fatty acids was significantly (*p* < 0.01) higher in 1.25% SRGF ASC vs. both 10% FBS and 5% SRGF ASC (Table 1, NS columns). Total monounsaturated fatty acid availability was significantly (*p* < 0.01) lower in 1.25% SRGF ASC vs. both 10% FBS and 5% SRGF ASC. The ratio between saturated and pooled unsaturated fatty acids was significantly (*p* < 0.01) higher in 1.25% SRGF ASC vs. both 5% SRGF and 10% FBS ASC. The ratio between saturated and pooled unsaturated fatty acids was not correlated to NF efficiency (R^2^ = 0.1) of our ASC.

Pooled n-3 polyunsaturated fatty acids were significantly (*p* < 0.01) lower in both 5% and 1.25% SRGF ASC vs. 10% FBS ASC, while n-6 polyunsaturated fatty acids were increased in 5% SRGF ASC vs. both 10% FBS and 1.25% SRGF ASC. The n-6 to n-3 ratio in 10% FBS ASC membranes was significantly (*p* < 0.01) lower vs. both 5% and 1.25% SRGF ASC. Then, independently from its availability in the cell culture medium, SRGF differentially affected n-6 to n-3 ratio in cell membranes when compared to 10% FBS. Moreover, in general, the n-6 to n-3 ratio displayed a tendency to be in direct correlation with NF efficiency (R^2^ = 0.89).

Table 1 shows percent availability of pooled fatty acids evaluated in membranes of 10% FBS ASC and 5% and 1.25% SRGF ASC. Lipids were divided in classes of saturated fatty acids (SFA), mono-unsaturated fatty acids (MUFA), and poly-unsaturated fatty acids (PUFA). PUFA were further divided in omega-6 (n-6) and omega-3 (n-3) fatty acid subgroups. Ratios between total SFA and unsaturated fatty acids (UFA), as well as between total n-6 and total n-3, were also added. UFA were calculated as the sum of MUFA and PUFA. NS, not supplemented; ACD-3, cells supplemented by ACD3 n-6 fatty acid mixture; ACD-4, cells supplemented by ACD4 n-3 fatty acid mixture. a, *p* < 0.05 vs. NS comparing cells expanded by the same medium additive. b, *p* < 0.01 vs. 10% FBS NS. c, *p* < 0.01 vs. 5% SRGF NS. d, *p* < 0.01 vs. 1.25% SRGF NS.

**Figure 5:**
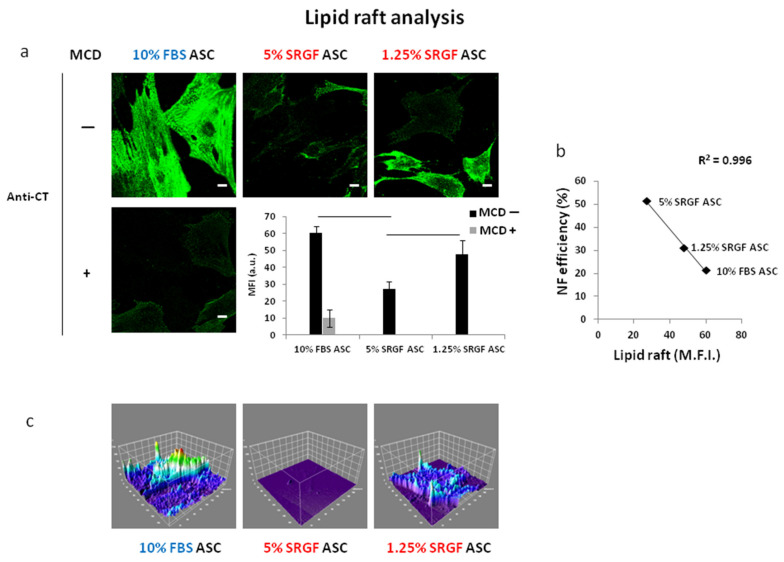
(**a**) shows representative images 10% FBS and 5% SRGF and 1.25% SRGF ASC labelled by fluorescent anti-CT antibodies (see Methods) to detect lipid raft availability on membranes. Methyl-β-cyclodextrin (MCD), a cholesterol scavenging compound, was used at sublethal concentration as technical control for assay specificity and it was applied only on 10% FBS ASC. Evaluation of mean fluorescence intensity as arbitrary units (M.F.I. (a.u.)) was also reported. (**b**) reports the correlation between lipid raft availability on cell membrane and NF efficiency evaluated in 10% FBS and 5% and 1.25% SRGF ASC. Solid lines connect significantly (*p* < 0.01) different mean values. Scale bars 5 μm. (**c**) reports a 3D surface plot analysis (ImageJ) graphically describing lipid raft signal intensity and distribution in single cells along the acquired z-stack. Reported images and (mean) quantification values represent results derived from at least three independent experimental tests.

To further characterize membrane properties of our ASC, we analyzed lipid raft distribution on the cell surface. Raft labelling was performed by a fluorescent antibody recognizing binding of exogenous cholera toxin to lipid rafts themselves. To demonstrate assay sensitivity (Figure 5a), we showed strong fluorescent signal reduction after addition of a sublethal dose of the cholesterol and lipid raft scavenging agent MCD. As displayed in Figure 5a, lipid raft density in membranes of 10% FBS ASC was significantly (*p* < 0.01) higher than in 5% SRGF ASC. In turn, lipid raft abundance in 1.25% SRGF ASC was shown to be significantly (*p* < 0.01) higher than in 5% SRGF ASC. Interestingly, (Figure 5b) we could demonstrate a significant inverse correlation between lipid raft availability on cell membrane and NF efficiency evaluated in 10% FBS and 5% and 1.25% SRGF ASC.

### 3.5. n-3 or n-6 Fatty Acid Supplementation and MCD Addition at Appropriate Concentrations

**Figure 6:**
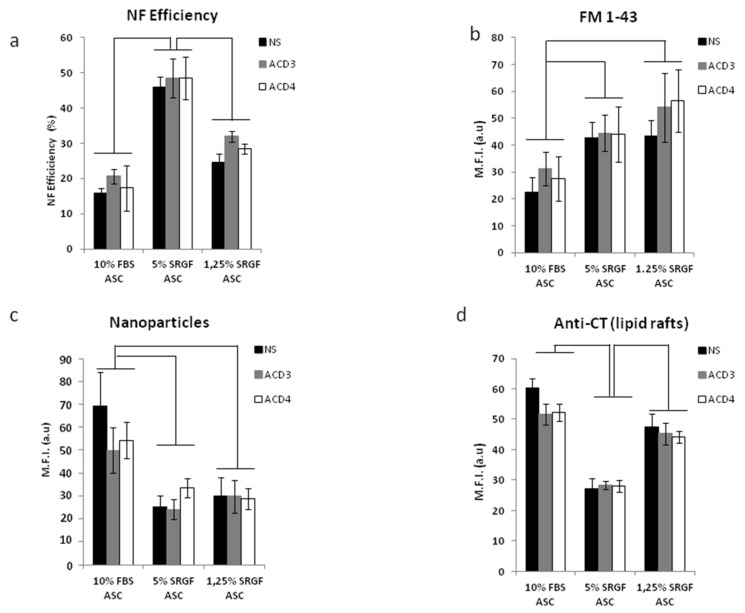
Figure 6 shows the impact of ACD3 (n-6 fatty acid mixture) and ACD4 (n-3 fatty acid mixture) supplementation on: NF efficiency (**a**), FM 1-43 uptake (**b**), nanoparticle incorporation (**c**), and lipid raft occurrence by anti-CT labelling (**d**). NS, not supplemented. Solid lines connect significantly different (*p* < 0.05) groups of means. Reported mean values are derived from at least three independent experimental tests.

To challenge the hypothesis that n-6 to n-3 ratio in cell membranes can affect NF efficiency as well as phagocytosis or endocytosis processes in our ASC, we supplemented n-3 or separately n-6 fatty acid mixtures for 24 h in cell culture media of 10% FBS and 5% and 1.25% SRGF ASC. As displayed in Table 1, supplementation with ACD3 (containing n-6 fatty acids) and ACD4 (containing n-3 fatty acids) increased availability of n-6 and n-3 fatty acids in cell membranes of all analyzed ASC (*p* < 0.05) (Table 1). When compared to unsupplemented controls, n-6/n-3 ratio was significantly decreased by ACD4 supplementation in 1.25% and 5% SRGF ASC. ACD3 supplementation significantly increased n-6/n-3 ratio in 10% FBS as well as in 1.25% and 5% SRGF ASC (Table 1). The ratio between saturated and unsaturated fatty acids (Table 1) was significantly affected by both ACD3 and ACD4 in 10% FBS ASC (increase) as well as in 5% SRGF (decrease) or 1.25% SRGF ASC (mild increase). Figure 6a shows that neither ACD3 nor ACD4 supplementation could affect NF efficiency when delivering the pMaxGFP vector to 10% FBS as well as to 5% and 1.25% SRGF ASC. Similarly, ACD3 and ACD4 supplementation failed to affect FM 1-43 (Figure 6b) as well as nanoparticle (Figure 6c) uptake in 10% FBS as well as in 5% and 1.25% SRGF ASC. Presence of lipid rafts on cell membranes of 10% FBS and 5% and 1.25% SRGF ASC was not affected by short term ACD3 or ACD4 supplementation (Figure 6d).

**Figure 7:**
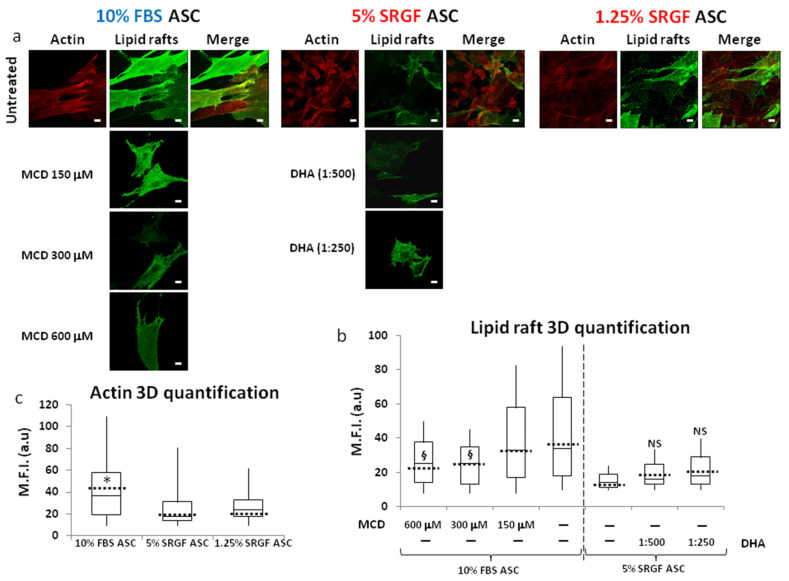
(**a**) reports representative images of 10% FBS and 5% and 1.25% SRGF ASC labelled to detect both total intracellular actin as well as lipid rafts. Moreover, (**a**) reports also images of lipid raft staining performed in 10% FBS ASC treated with non-toxic concentrations (150 μM, 300 μM, and 600 μM) Methyl-β-cyclodextrin (MCD). In parallel, images of lipid raft labelling performed in 5% SRGF ASC supplemented with higher (1:250) or lower (1:500) concentrations of doxosahexaenoic acid (DHA) were reported. Scale bars: 5 μm. Quantification of lipid raft related mean fluorescence intensity in each image of acquired z-stacks was reported as box plots in (**b**). Furthermore, 300 and 600 μM MCD addition to the cell culture medium mildly but significantly reduced lipid raft availability in 10% FBS ASC, while both DHA concentrations smoothly increased lipid rafts in 5% SRGF ASC. ^§^, *p* < 0.05 vs. unsupplemented 10% FBS ASC; NS, not significant vs. unsupplemented 5% SRGF ASC. Quantification of total actin related mean fluorescence intensity in each image of acquired z-stacks was reported as box plots in (**c**). Dashed black bars represent mean values. Total actin content in 5% and 1.25% SRGF ASC is significantly lower vs. 10% FBS ASC; *, *p* < 0.05 vs. both 5% and 1.25% SRGF ASC. Reported images and (mean) quantification values represent results derived from at least three independent experimental tests.

In additional experiments, recapitulating a previously published method [26], we added DHA only for a long duration (5 days) at non-toxic concentrations in the culture medium of 5% SRGF ASC. As displayed in Figure 7a,b, mean values of labelled lipid raft intensity showed a tendency to increase, but unfortunately statistical significance was not achieved. NF efficiency was not differently affected by DHA supplementation (data not shown).

In a further attempt to disentangle the possible influence of lipid rafts on NF efficiency in our expanded ASC, we exposed 10% FBS ASC cells to different MCD concentrations, tailored in order to minimize cell toxicity. As evidenced in Figure 7a,b, we observed a smooth but significant reduction in lipid raft occurrence as a consequence of 300 and 600 μM MCD addition to the cell culture medium. Nevertheless, in such experimental setting, we could not display a modulation of NF efficiency corresponding to induced lipid raft changes (data not shown).

To further characterize our differently expanded ASC, in the same experimental sessions, we additionally evaluated cellular content of total actin fibers and we demonstrated (Figure 7a,c) that both 5% and 1.25% SRGF ASC were characterized by lower intracellular actin availability when compared to 10% FBS ASC.

In addition, as displayed in Supplementary Figure S1, we could not observe relevant differences between 10% FBS and 5% SRGF ASC regarding total protein content, as well as membrane protein expression.

## 4. Discussion

Expanded MSC or ASC are promising advanced cellular therapy products in regenerative medicine [37] as well as in cancer patients [38]. Genetic engineering of such cells is required to improve and tailor their therapeutic potential [39,40]. While viral genetic modification approaches are plagued by potential safety drawbacks, traditional non-viral techniques of gene delivery to ASC are safer and easier to apply [14]. Such cell modification approaches could contribute to improve biological activity of MSC/ASC and to ameliorate the safety profile of such cell products when applied in advanced therapy protocols. Exogenous DNA can be delivered by NF to expanded MSC with sufficient efficiency [15,16,17,41] and NF is compatible with GMP guidelines [42]. Amelioration of NF efficiency is required, but parameters affecting NF efficiency in MSC or ASC are poorly characterized. In this work, we investigated the role of GMP compatible ASC expansion conditions on NF efficiency. In particular, ASC were cultured in the presence of SRGF, a clinical-grade medium additive for ASC ex vivo expansion. We previously showed that 5% SRGF addition to the ASC culture medium can lead to rapid cell proliferation, maintaining cell biological properties and karyotype stability [11]. In the same study, we showed that expression of immunophenotype markers of SRGF expanded cells was fully compatible with criteria defined by the International Society for Cellular Therapy [33]. In the present study, NF was performed in SRGF expanded ASC using a commercially available device with dedicated single use kits [17,41,42]. We identified appropriate NF conditions that, in our 5% SRGF expanded ASC, allowed higher transfection efficiency coupled with increased cell viability, in turn leading to a significantly elevated yield of modified ASC. A different program was previously [17,41] defined as suitable for the ASC transfection; such discrepancy can be ascribed to the different cell buffer used for electric shock administration, as well as to MSC origin [41,43] or to the cell donor age [17]. Here, we also showed that increasing SRGF availability in cell expansion medium over 5% failed to provide beneficial effects on cell transfection performance. These results are coherent with our previous observations showing that increased SRGF concentrations in culture medium over 5% failed to proportionally improve ASC proliferation rate [44]. We can hypothesize that an addition of 5% SRGF to the growth medium provides saturating availability of factors influencing not only cell proliferation rate, but also NF efficiency. Interestingly, we also demonstrated that reducing SRGF concentrations in culture medium at or below 1.25% caused NF efficiency to lower. Thus, we can conclude that the addition of 5% SRGF to the culture medium is the most appropriate GMP compatible approach, both for cell expansion and to obtain an abundant amount of genetically modified cells using a safe non-viral approach such as NF. This represents a remarkable result, helping translation of basic science achievements to the manufacturing process of a clinically relevant cellular product, modified by a non-viral approach.

Expanded ASC were previously transfected by a different transfection system as microporation, with almost 50% efficiency and a satisfactory viability [15], but such an approach was potentially not compatible with GMP guidelines. The impact of cell culture protocol on transfection performance was only previously explored in part; fibroblast growth factor 2 supplementation was associated with increased NF efficiency in ASC, while human serum addition reduced the fraction of transfected cells [17,45]. In our SRGF expanded ASC, the NF efficiency was far higher than in other studies [46], adopting the same NF device but different cell electroporation buffers. NF efficiency was presently evaluated using a commercial empty vector encoding for GFP; to validate our results, we also showed that the NF protocol can be applied to 5% SRGF ASC to abundantly overexpress an endogenous protein. Independently from the DNA delivery method, to evaluate transfection efficiency while overexpressing a desired gene (i.e., not only a reporter/tag as GFP), the possible confounding effect of protein toxicity must be avoided. Principally, for such reasons, we selected CD63 as target gene to validate our NF approach. Changes in the expression of such tetraspanin were not previously shown to decrease cell viability or to deregulate cell cycle arrest or apoptosis [47]. CD63 is involved in microvesicle formation [47] but such feature should not have influenced the internalization process of NF, as protein overexpression occurs after the electroporation program is applied.

Previous works demonstrated that electroporated cells could efficiently proliferate without modification of surface marker expression and of differentiation or clonogenic capacity [17,41]. For such reasons, identity and potency assays were not performed in our GFP transfected ASC. However, we observed that growth rate of 5% SRGF expanded ASC after NF was aligned with non-transfected ASC counterpart [11] or with MSC previously expanded in xeno-free media [48]. Moreover, as previously published [12], when compared to flatter 10% FBS ASC, 5% SRGF ASC maintained their evident spindle-shaped morphology, linked to higher proliferation rate.

In order to confirm that cell culture conditions can actually affect routes of cell trafficking across the membrane, we also investigated SRGF impact on other cellular transport pathways, as vesicle endocytosis or phagocytosis. Even though nanoparticle phagocytosis and vesicle endocytosis can be used as DNA delivery routes [49,50], direct assessment of vector uptake efficiency by such means was not our aim, thus GFP encoding DNA was not administered. Only 10% FBS ASC could efficiently uptake relevant amounts of nanoparticle aggregates, while the pool of internal vesicles labelled by FM 1-43 dye was shown to be considerably abundant only in SRGF expanded ASC. This evidence suggests that SRGF can selectively modulate separate cellular processes of internalization.

Besides the evidence discussed above, in this work we also attempted to define mechanisms potentially explaining SRGF mediated impact on NF efficiency of ASC. Biochemical processes leading to pore formation in the cell membrane after electric pulse are extremely complex and they are not, to date, fully clarified [18,19]. Thus, in a first attempt to characterize relevant parameters potentially affecting NF efficiency in our ASC, we evaluated fatty acid membrane composition. Physical properties of cell membrane could affect gene internalization efficiency; in prokaryotic cells, increased membrane fluidity was shown to improve pore formation rate upon electric shock [51]. On the other hand, conflicting results suggested that reversible electroporation rate in eukaryotic cell lines was not linked to their membrane fluidity [21,22]. Enriched availability of saturated fatty acids normally increases membrane stiffness, while unsaturated fatty acids improve membrane fluidity [23,52]. Notably, in our results, the baseline ratio between saturated and unsaturated fatty acids was not associated with NF efficiency changes.

As previous works showed that n-6 to n-3 ratio was far higher in cells expanded in presence of human serum when compared to FBS [53], we also evaluated availability of n-6 and n-3 lipids in membranes of our ASC. SRGF is, in fact, a hemoderivative, and dietary habits of western Europe are likely to determine a high n-6 to n-3 ratio in its lipid composition [54]. Both n-6 and n-3 polyunsaturated fatty acids can affect membrane properties [55] and downstream cell signaling [56,57,58]. In particular, n-3 and n-6 fatty acids are known to play, respectively, an anti- and pro-inflammatory role [55,57], affecting synthesis rate of eicosanoids [59]. Thus, we hypothesized n-6 to n-3 ratio in cell membranes could affect pore formation rate after electric shock administration. Interestingly, we observed a strikingly enhanced n-6 to n-3 polyunsaturated fatty acid ratio in efficiently electroporated cells as 5% and 1.25% SRGF ASC. Moreover, the n-6 to n-3 ratio was shown to be inversely related to the phagocytic activity of specialized cells [60]; coherently, in this work, decreased n-6 to n-3 ratio was demonstrated in 10% FBS ASC which showed enhanced phagocytosis properties. In the attempt to challenge the role of n-6 to n-3 fatty acid ratio changes on NF efficiency, as well as on phagocytosis or on vesicle internalization, we supplemented our ASC with ACD3 and ACD4, as mixtures containing n-6 and n-3 fatty acids, respectively. Due to induced cell toxicity (data not shown), ASC supplementation by ACD3 and ACD4 was performed only at short term, i.e., 24 h. While the n-6 to n-3 ratio in our ASC membranes was actively modified by both supplements, we could not show effects either on NF or phagocytosis as well as on vesicle internalization efficiency. Such results would suggest that modulation of NF efficiency in our expanded ASC is not based on acute variations of n-6 to n-3 fatty acid ratio.

On the other hand, after an electric pulse, pore formation in cell membranes was previously shown to preferentially occur in liquid disoriented regions of the membrane, i.e., outside the so called lipid rafts [20]. For such reasons, we labelled and quantified occurrence of membrane lipid rafts. Interestingly, poorly transfected 10% FBS ASC exhibited a higher lipid raft availability on their membranes when compared to efficiently electroporated SRGF ASC. Increased lipid rafts on 10% FBS ASC surface is likely to reduce the relative fraction of fluid and liquid disoriented membrane [61], thus hampering the probability of pore formation after an electric pulse. This hypothesis is in part supported by the linear inverse correlation we showed between lipid raft levels on cell membranes and NF efficiency in differently expanded ASC. Moreover, such results obtained in living cells are aligned with previous evidences obtained by taking advantage of artificial membranes [20].

Notably, nanoparticles phagocytosis is known to occur through lipid raft associated mechanisms [29,30,62], while fluid internalization through vesicle endocytosis is likely to occur in membrane domains not containing lipid rafts [63]. Thus, higher lipid raft exposure on 10% FBS ASC membranes is reasonably in accordance with their increased phagocytosis capacity, while higher vesicle internalization capacity of 5% and 1.25% SRGF ASC can be explained by their poor lipid raft availability. Changes in vesicle endocytosis capacity were coherent with changes in NF efficiency levels. Thus, present results suggest that, in our ASC, lipid rafts could facilitate nanobeads uptake by phagocytosis, otherwise acting as a barrier limiting endocytosis and electric shock induced pore formation for subsequent DNA uptake.

In order to challenge the hypothesis that SRGF mediated lipid raft reduction can increase NF efficiency, we attempted to modulate lipid raft occurrence on cell membranes by alternative approaches. Concentrations of used reagents were tailored to limit cell toxicity that could obviously prevent successful NF of our ASC. As discussed above, acute modulation of n-3 or n-6 fatty acids failed to affect lipid raft abundance on cell membrane; unfortunately, prolonged supplementation of such fatty acid was not feasible due to cell toxicity. Otherwise, prolonged supplementation of DHA in permissive conditions for ASC growth did not induce the expected increase in lipid rafts of 5% SRGF ASC [26]. Moreover, adding low concentrations at longer terms of the cholesterol scavenging agent MCD, we could induce only a mild reduction in lipid raft density in 10% FBS ASC. Unfortunately, no significant changes in NF efficiency could be displayed in association with such partial lipid raft modulation; we can hypothesize that present MCD mediated lipid raft changes were not sufficient to modulate NF efficiency as biological effect. Attempts to further increase MCD and DHA concentrations in order to amplify lipid raft modulation extent seriously damaged cell integrity. Thus, despite our efforts, obtained results are not sufficient to draw a clear conclusion about lipid raft role in such a complex and partially unknown process as NF. Nevertheless, considering previously published results [20] and the significant correlation we observed between lipid raft levels and NF efficiency, we cannot exclude lipid raft could play a role in the NF process.

In a previously published work [64], cytoskeletal weakening had a protective effect that enabled higher intracellular uptake and less cell viability loss after transient pore formation by laser irradiation. In a final attempt to deepen our investigation, we showed that efficiently transfected 5% and 1.25% SRGF ASC were characterized by lower actin content. Such evidence is aligned with the previously published work [64], despite differences in the transient cell permeabilization approach.

## 5. Conclusions

We demonstrated that presence of a GMP compatible medium additive as SRGF in ASC culture medium can improve efficiency of NF, i.e., a non-viral approach of gene transfection. This can pave the way to safer and effective genetic modification approaches for clinical applications. Nevertheless, this preclinical study cannot exclude that cell electroporation could induce additional and undesired drawbacks on modified ASC; studies in animal models and subsequent controlled clinical trials will contribute to shed light on this important safety issue. Moreover, as a second aim of the work, we also tried to identify principal mechanisms accounting for observed effects. We showed that SRGF mediated increase in NF efficiency was accompanied by coincident lipid composition changes of the cell membrane, in terms of increased n-6 to n-3 ratio. Moreover, inverse correlation between lipid raft availability and NF efficiency was shown. Thus, such parameters could potentially be involved in NF efficiency regulation. We admit that attempts to define the net independent contribution of selected parameters on NF efficiency were not successful; for such reasons, clear and safe conclusions could not be drawn. We also showed that, beside influencing membrane composition, SRGF can also reduce total actin content. We can only hypothesize that the simultaneous modification of different cellular features could be required to actually induce changes in NF efficiency. In this work, NF was only actively affected by the SRGF, which is an extremely complex mixture of biochemical compounds [10] with composite impact on cell physiology. Required additional investigations regarding this issue are extremely challenging considering (i) drawbacks on cell viability mediated by needed reagents for selective modulation of membrane composition and (ii) lack of deep knowledge about parameters generally determining pore formation on cell membrane upon electric pulse.

## 6. Patents

We disclose that the approach comprising appropriate cell expansion conditions and NF protocol was submitted for patenting (Application number 102020000030692, Submission date: 14 December 2020)

## Figures and Tables

**Table 1:** ASC lipid membrane composition.

	10% FBS ASC			5% SRGF ASC			1.25% SRGF ASC		
	NS	ACD3	ACD4	NS	ACD3	ACD4	NS	ACD3	ACD4
SFA	44.7 ± 0.7	68.6 ± 7.3 ^a^	66.6 ± 4.8 ^a^	48.6 ± 1.6	60.4 ± 4.2 ^a^	63.4 ± 4.6 ^a^	72.4 ± 2.7 ^b.c^	65.0 ± 3.2	68.1 ± 1.5
MUFA	26.6 ± 1.0	9.4 ± 3.1 ^a^	13.3 ± 3.3 ^a^	24.4 ± 1.5	9.8 ± 1.6 ^a^	11.7 ± 2.7 ^a^	12.8 ± 1.6 ^b.c^	6.8 ± 0.7 ^a^	7.6 ± 0.1 ^a^
n-3 PUFA	7.9 ± 0.3	2.1 ± 0.7 ^a^	10.1 ± 2.1 ^a^	1.5 ± 0.1 ^b^	0.9 ± 0.3	14.7 ± 0.9 ^a^	1.4 ± 0.3 ^b^	1.1 ± 0.8	16.4 ± 0.7 ^a^
n-6 PUFA	15.3 ± 0.7	20.0 ± 4.9 ^a^	10.0 ± 1.4 ^a^	22.3 ± 1.3 ^b.d^	29.0 ± 4.1 ^a^	10.2 ± 2.1 ^a^	13.4 ± 1.0	27.1 ± 5.0 ^a^	7.9 ± 1.2 ^a^
SFA/UFA	0.9 ± 0.2	2.2 ± 0.6 ^a^	2.0 ± 0.4 ^a^	1.0 ± 0.8	1.9 ± 0.7 ^a^	2.1 ± 0.9 ^a^	2.6 ± 0.3 ^b^	1.9 ± 0.3 ^a^	2.1 ± 0.4 ^a^
n-6/n-3	1.9 ± 0.2	9.5 ± 2.1 ^a^	1.0 ± 0.1	14.3 ± 3.5 ^b^	33.1 ± 7.1 ^a^	0.7 ± 0.1 ^a^	9.7 ± 3.0 ^b^	24.0 ± 6.1 ^a^	0.5 ± 0.3 ^a^

## Data Availability

All data and materials were included in the present work. Details could be obtained upon reasonable request to the corresponding author.

## Supplementary Materials

The following are available online at https://www.mdpi.com/article/10.3390/cells10123412/s1, Supplementary Figure S1. The figure reports original unadjusted and unmodified images of CD63 and GAPDH Western blots that were enclosed in Figure 2b. In Figure 2b, loading gel order was flipped to consistently show, from left to right, 10% FBS ASC and 5% SRGF ASC, as in other figures. Supplementary Figure S2 reports detailed and higher magnification images of 10% FBS and 5% SRGF ASC after exposure to 50 nm and 120 nm nanobeads to assess phagocytosis potential (Figure 4). Internalized nanoparticles are evidenced by white arrows. Fluorescence cell imaging (20× objective) was performed setting conditions (lamp output energy: 75%; exposure time: 160 msec-green channel for CFDA-SE, 80 msec-red channel for nanobeads; digital gain: 1; offset: default) in untreated cells in order to fully avoid cell autofluorescence (dark pictures). Supplementary Figure S3. Figure S3 shows whole cell and membrane protein level comparison between 10% FBS and 5% SRGF ASC. Graphical abstract was created in BioRender.com (accessed on 26 November 2021). Supplementary Table S1: Detailed analysis of selected fatty acid membrane composition in 10% FBS and 5% and 1.25% SRGF ASC.
