# Supplementary material for: Nucleofection of Adipose Mesenchymal Stem/Stromal Cells: Improved Transfection Efficiency for GMP Grade Applications
Source: Cells. 2021 Dec 3;10(12):3412. doi: 10.3390/cells10123412 (PMC8700287; doi:10.3390/cells10123412)
Supplement: Supplementary file 1 [file cells-10-03412-s001.zip › Supplementary file.pdf]

Supplementary Table S1: Detailed analysis of selected fatty acid membrane composition in 10% FBS and 5% and 1.25% SRGF ASC.

	10% FBS ASC			5% SRGF ASC			1.25% SRGF ASC		
**IUPAC**	NS	ACD3	ACD4	NS	ACD3	ACD4	NS	ACD3	ACD4
14:00	1.5±0.1	1.5±0.1	1.4±0.1	1.8±0.1	1.4±0.1	1.4±0.1	1.7±0.1	1.6±0.2	1.8±0.1
16:00	21.0±0.4	27.0±2.0	26.6±2.0	24.3±0.7	25.8±0.1	26.7±0.1	29.2±1.1	25.1±1.3	27.0±0.4
18:00	22.3±0.2	40.1±5.2	38.6±2.7	22.5±0.8	33.2±4.2	35.3±4.4	41.5±1.7	38.2±1.7	39.2±1.0
16:1n-7	1.1±0.2	0.2±0.1	0.1±0.1	1.0±0.2	0.3±0.1	0.3±0.1	0.2±0.1	0.3±0.1	0.2±0.1
18:1n-9	25.1±0.5	7.8±2.9	11.5±2.9	22.7±0.8	8.3±1.4	10.1±2.3	10.8±1.5	5.0±0.3	5.8±0.1
20:1n-9	0.4±0.3	1.4±0.3	1.8±0.3	0.6±0.5	1.2±0.2	1.2±0.2	1.8±0.1	1.5±0.4	1.6±0.1
18:3n-3	0.2±0.1	0.4±0.1	3.3±0.6	0.1±0.1	0.3±0.1	5.2±0.1	0.5±0.1	0.2±0.2	9.0±0.1
20:5n-3	0.4±0.1	0.1±0.1	1.5±0.3	0.1±0.1	0.1±0.1	3.5±0.2	0.1±0.01	0.1±0.1	2.9±0.1
22:5n-3	3.4±0.1	0.9±0.3	2.8±0.6	0.5±0.1	0.4±0.2	2.6±0.3	0.7±0.1	0.5±0.3	2.0±0.2
22:6n-3	4.1±0.1	0.8±0.3	2.6±0.5	0.9±0.1	0.2±0.1	3.4±0.3	0.2±0.2	0.4±0.2	2.5±0.2
20:4n-6	7.7±0.3	4.7±1.8	2.1±0.5	7.4±0.2	6.6±1.2	1.4±0.5	2.9±0.3	5.0±0.8	0.8±0.1
18:2n-6	1.6±0.1	4.7±1.1	4.0±0.1	7.7±0.6	9.7±0.7	5.5±0.9	4.6±0.1	9.4±2.6	3.6±1.0
18:3n-6	0.3±0.1	0.2±0.1	0.2±0.1	0.3±0.1	0.5±0.3	0.3±0.1	0.6±0.1	0.6±0.3	0.2±0.1
20:3n-6	2.1±0.1	6.7±1.6	1.2±0.1	3.4±0.1	8.1±1.3	1.2±0.2	1.7±0.3	9.1±1.0	1.1±0.1
22:4n-6	3.2±0.1	2.8±0.4	1.5±0.1	2.5±0.2	3.3±0.5	1.1±0.2	2.6±0.1	2.3±0.1	1.2±0.1
22:5n-6	0.3±0.1	0.15±0.1	0.1±0.1	0.3±0.1	0.1±0.1	0.1±0.1	0.3±0.1	0.2±0.1	0.2±0.1
20:2n-6	0.1±0.1	0.7±0.2	0.7±0.1	0.7±0.2	0.6±0.1	0.6±0.1	0.8±0.1	0.7±0.1	0.7±0.1

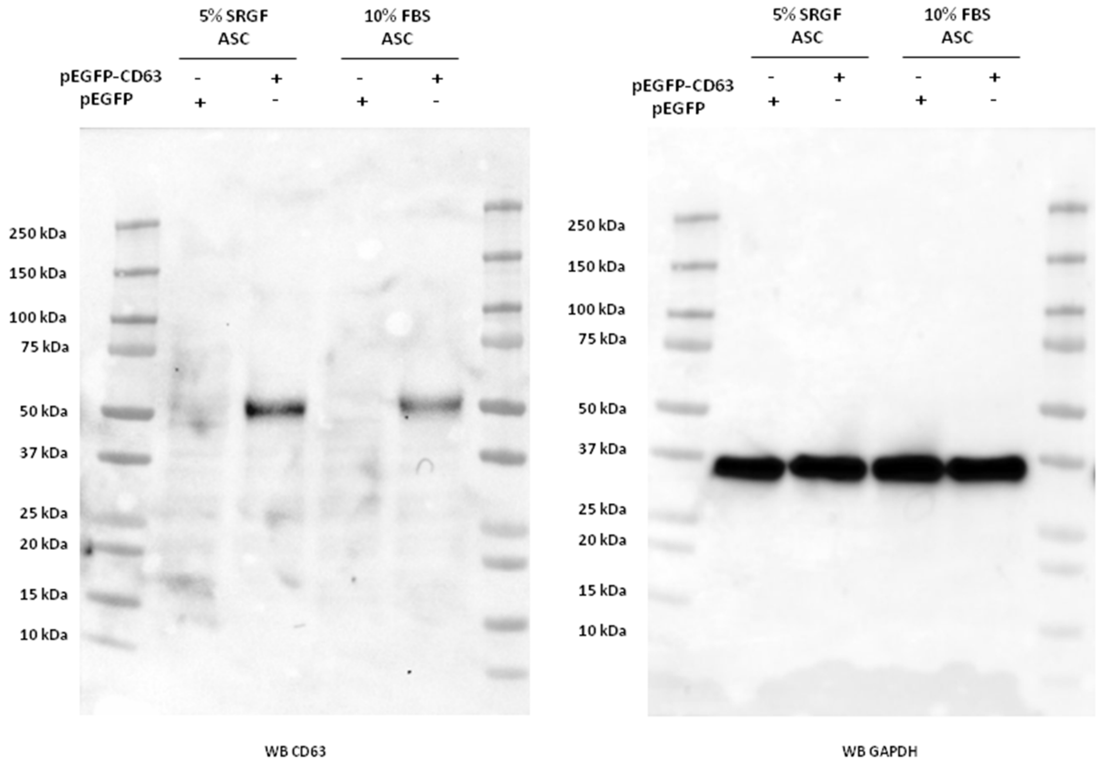

Supplementary Figure S1. The figure reports original unadjusted and unmodified images of CD63 and GAPDH Western blots that were enclosed in Figure 2b. In Figure 2b, loading gel order was flipped to consistently show, from left to right, 10% FBS ASC and 5% SRGF ASC, as in other figures.

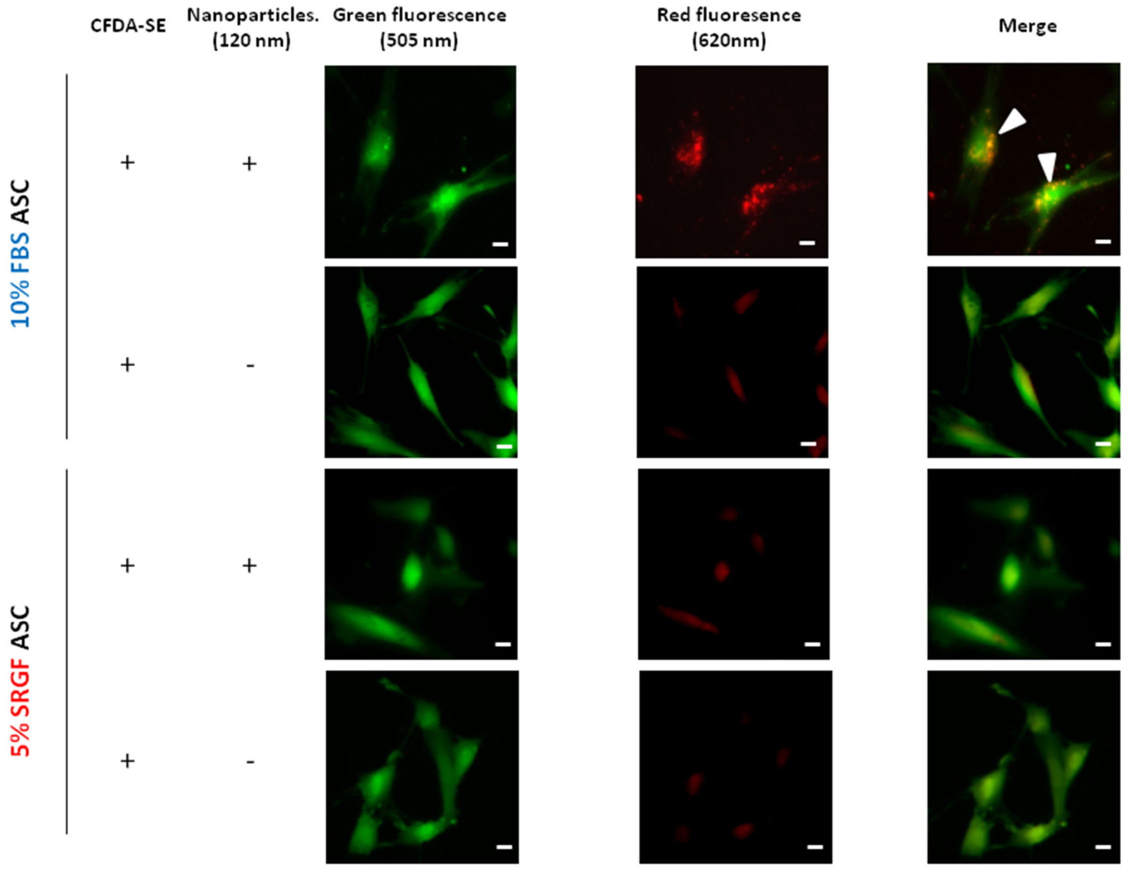

Supplementary Figure S2. reports detailed and higher magnification images of 10% FBS and 5% SRGF ASC after exposure to 50 nm and 120 nm nanobeads to assess phagocytosis potential (Figure 4). Internalized nanoparticles are evidenced by white arrows. Fluorescence cell imaging (20× objective) was performed setting conditions (lamp output energy: 75%; exposure time: 160 msec-green channel for CFDA-SE, 80 msec-red channel for nanobeads; digital gain: 1; offset: de-fault) in untreated cells in order to fully avoid cell autofluorescence (dark pictures).

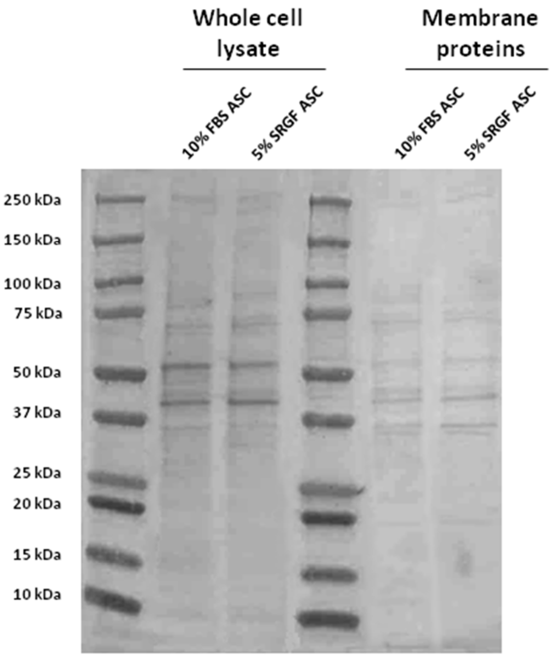

Supplementary Figure S3. Figure S3 shows whole cell and membrane protein level comparison between 10% FBS and 5% SRGF ASC. Graphical abstract was created in BioRender.com (accessed on 26th of November, 2021). Supplementary Table S1: Detailed analysis of selected fatty acid membrane composition in 10% FBS and 5% and 1.25% SRGF ASC.
